# Phage-Resistant Phase-Variant Sub-populations Mediate Herd Immunity Against Bacteriophage Invasion of Bacterial Meta-Populations

**DOI:** 10.3389/fmicb.2019.01473

**Published:** 2019-07-05

**Authors:** Christopher J. R. Turkington, Andrew Morozov, Martha R. J. Clokie, Christopher D. Bayliss

**Affiliations:** ^1^Department of Genetics and Genome Biology, University of Leicester, Leicester, United Kingdom; ^2^Department of Mathematics, University of Leicester, Leicester, United Kingdom

**Keywords:** phase variation, localised hypermutation, bacteriophage, *Haemophilus*, herd immunity, phage resistance, phage receptor

## Abstract

Hypermutable loci are widespread in bacteria as mechanisms for rapid generation of phenotypic diversity within a population that enables survival of fluctuating, often antagonistic, selection pressures. Localized hypermutation can mediate phase variation and enable survival of bacteriophage predation due to high frequency, reversible alterations in the expression of phage receptors. As phase variation can also generate population-to-population heterogeneity, we hypothesized that this phenomenon may facilitate survival of spatially-separated bacterial populations from phage invasion in a manner analogous to herd immunity to infectious diseases in human populations. The *lic2A* gene of *Haemophilus influenzae* is subject to “ON” and “OFF” switches in expression mediated by mutations in a 5′CAAT repeat tract present within the reading frame. The enzyme encoded by *lic2A* mediates addition of a galactose moiety of the lipopolysaccharide. This moiety is required for attachment of the HP1C1 phage such that the ON state of the *lic2A* gene is associated with HP1c1 susceptibility while the OFF state is resistant to infection. We developed an “oscillating prey assay” to examine phage spread through a series of sub-populations of *Haemophilus influenzae* whose phage receptor is in an ON or OFF state. Phage extinction was frequently observed when the proportion of phage-resistant sub-populations exceeded 34%. *In silico* modeling indicated that phage extinction was interdependent on phage loss during transfer between sub-populations and the frequency of resistant sub-populations. In a fixed-area oscillating prey assay, heterogeneity in phage resistance was observed to generate vast differences in phage densities across a meta-population of multiple bacterial sub-populations resulting in protective quarantining of some sub-populations from phage attack. We conclude that phase-variable hypermutable loci produce bacterial “herd immunity” with resistant intermediary-populations acting as a barricade to reduce the viral load faced by phage-susceptible sub-populations. This paradigm of meta-population protection is applicable to evolution of hypermutable loci in multiple bacteria-phage and host-pathogen interactions.

## Introduction

Hypermutable loci as mediators of survival against constantly fluctuating selection pressures are a predictable outcome of the evolution of evolvability as stated in the Red Queen hypothesis (van Valen, 1973; Moxon et al., 1994). Fluctuating selection pressures are regularly faced by bacteria during persistence in human hosts, where bacteria adhere to host surfaces while contending with varying nutrient concentrations, frequent exposure to immune effectors, and predation by bacteriophages. These fluctuations often select for and against opposing gene expression states of single loci leading to evolution of localized hypermutable mechanisms that produce frequent switches in single-gene expression states.

One class of hypermutable loci facilitate survival of fluctuations in conflicting selective pressures by pre-emptive, frequent, and reversible generation of “ON/OFF” adaptive variants, in a process known as “phase variation” (PV; Moxon et al., 1994; van der Woude and Bäumler, 2004). A major mechanism of PV involves increases and decreases in identical, tandemly-arranged DNA repeats (microsatellites) by slipped strand mispairing during DNA replication (Moxon et al., 2006; Bayliss, 2009). The obligate human respiratory commensal and pathogen *H. influenzae* contains an expansive array of repeat driven phase variable loci (Power et al., 2009). Several of these loci are required for addition of sugar molecules onto the surface-exposed outer-core of the lipooligosaccharide (LOS).

Bacteriophages are major predators of all species of bacteria. Bacteriophages attach to a specific host receptor with attachment triggering a process of cell invasion that can lead to phage replication or, if it is a lysogenic phage, invasion of the bacterial chromosome. Phage replication leads to production of multiple phage particles per bacterial cell with this number usually being within a specific range defined as the burst size. This amplification of phage particles enables spread to neighboring cells within a bacterial population and to nearby populations within a meta-population (e.g., multiple colonies on an agar plate or multiple microcolonies on a mucosal surface). Individual bacterial cells or populations are described as being susceptible (termed S herein) to infection if they express the phage receptor and are able to support efficient phage replication. A resistant state (termed R herein) develops if by mutation the bacterial cell loses the phage receptor or otherwise restricts phage replication by, for example, acquiring a restriction-modification (RM) system that is active against the phage.

The development of phage resistance is known to occur by PV of either the receptor or an RM system for several phage-bacteria interactions in multiple bacterial species (Sørensen et al., 2011; Cota et al., 2012; Seed et al., 2012; Anjum et al., 2016) and has been observed for the HP1c1 infections of *H. influenzae* strain Rd (Zaleski et al., 2005). PV of the UDP-galactose-LOS-galactosyltransferase encoding gene, *lic2A*, is mediated by a 5'CAAT repetitive tract present in the open-reading frame (High et al., 1993). Phage HP1c1 attaches to an LOS epitope of *H. influenzae* strain Rd that contains the galactose sugar added by *lic2A* (Zaleski et al., 2005). PV of *lic2A* causes switching between phage susceptible (*lic2A* ON; the S state for this system) and phage resistant states (*lic2A* OFF; the R state). Partial resistance to phage HP1c1 in strain Rd is also mediated by PV of a Type I RM system (Zaleski et al., 2005).

While phage-receptor PV prevents viral propagation in individual populations, the frequency and distribution of resistant variants within larger meta-populations may also impose inhibitory effects on phage spread through multiple spatially-linked sub-populations. In order to explore this potential benefit, we examined how diversity in phage resistance/susceptible phenotypes generated by one hypermutable locus, *lic2A*, alters spread of phage HP1c1.

## Materials and Methods

### Phage and Bacterial Strains

Phage HP1c1, and the *lic2A* ON (Rd 30S) and OFF (Rd 30R) phase variants of *H. influenzae* were obtained from A. Piekarowicz (University of Warsaw, Poland). Phage HP1c1 is maintained in the lysogenic state within *H. influenzae* RM118-L. *H. influenzae* strains were cultured overnight at 37°C on 1% BHI agar supplemented with 10% Levinthal's supplement and 2 μg/mL nicotinamide adenine dinucleotide (NAD) or in 10 mL BHI broth supplemented with 2 μg/mL NAD and 10 μg/mL hemin (sBHI).

### Phage HP1c1 Stocks

Mitomycin C was added to a concentration of 300 ng/mL to an OD_600_ 0.1 culture of RM118-L in 10 ml of sBHI. After incubation for 8 h, the culture was centrifuged (4,946 × *g*, 4°C, 10 min) and then the supernatant was passed through a 0.22 μm syringe filter to obtain a phage suspension, which was stored at 4°C.

High titer phage stocks were propagated in *H. influenzae* Rd 30S by adding 100 μL of induced phage to mid-log phase cultures diluted to an OD_600_ of 0.01 in 10 ml sBHI and incubating for 8 h. Cultures were processed as described above.

### Determination of Phage Titers

Phage titers were determined using the small-drop plating assay (Mazzocco et al., 2009). Briefly, 150 μl of a mid-log phase Rd 30S culture, OD_600_ 0.1, was added to 3 mL of 0.3 % BHI agar (supplemented with 2 μg/mL NAD and 10 μg/mL hemin), mixed by inversion, and poured onto 1% BHI agar plates supplemented with 10% Levinthal's media and 2 μg/mL NAD. Ten-fold serial dilutions were spotted in triplicate 10 μL drops onto the soft agar (the minimum detection threshold is 33.3 PFU/mL).

### One-Step Growth Curve of Phage HP1c1

Overnight culture of either Rd 30S or Rd 30R were sub-cultured to OD_600_ 0.1 in 40 mL sBHI. Phage HP1c1 was added to a final titer of 1 ×10^5^ PFU mL^−1^ (MOI 0.001) and, after mixing, 1 mL was removed, passed through a 0.22 μm filter and used to determine the initial phage titer. The remaining suspension was incubated at 37°C for 10 min to allow phage to bind cells, after which the culture was centrifuged at 4,696 × *g* for 5 min at 4°C. The supernatant was discarded and the bacteria/bound phage pellet was resuspended in 40 mL fresh sBHI pre-chilled to 4°C. Centrifugation and washing in chilled broth was repeated again. After this, the bacteria/phage pellet was resuspended in 40 mL room temperature sBHI and aliquoted into 2 mL aliquots. These aliquots were incubated at 37°C with shaking at 100 rpm. Aliquots were removed for phage titration at 5 min intervals over 60 min.

### Phage HP1c1 Adsorption Assay

HP1c1 adsorption rate was measured as previously described (Kropinski, 2009). Rd 30S was sub-cultured in 10 mL sBHI to OD_600_ 0.1. A 1 mL aliquot was removed and used to determine bacterial cfu/mL by spreading 100 μL of 10-fold serial dilutions onto BHI 1% agar supplemented with 10% Levinthal's supplement and 2 μg mL^−1^ NAD and incubated overnight at 37°C. To the remaining 9 ml culture, 2 μL of 30 mg mL^−1^ chloramphenicol was added preventing phage replication during the assay. The culture was incubated at 37°C, with agitation at 100 rpm, for 5 min to allow the suspension to reach 37°C before phage addition followed by addition of 1 mL of a pre-warmed HP1c1 suspension to a final titer of ~1 ×10^7^ PFU mL^−1^. At 10 min intervals 50 μL of the suspension was removed and added to ice-chilled tubes containing 950 μL sBHI broth plus three drops of chloroform with these tubes then briefly vortexed, and placed back on ice until completion of the experiment. Phage titer was determined for each tube by adding 100 μL of the phage suspension to 150 μL of OD_600_ 0.1 Rd 30S, followed by addition of 3 mL BHI 0.3% agar supplemented with hemin and NAD. After mixing by inversion, the mixture was spread onto a 1% supplemented-BHI agar plate, allowed to set, incubated overnight at 37°C and then plaques were counted.

### Linear Oscillating Prey Assay

Two phase variants, namely, Rd 30S (*lic2A* ON, phage susceptible variant) and Rd 30R (*lic2A* OFF, phage resistant variant) were utilized for the linear oscillating prey assay. The *H. influenzae* Rd 30S and Rd 30R variants were prepared for each cycle of the assay by sub-culturing as described above in 20 mL of sBHI to an OD_600_ of 0.1. The first cycle of each linear series was initiated by inoculating a 5 mL aliquot of the relevant strain with HP1c1 at MOI ~0.01. Cultures were adjusted to 6 mL, mixed by inversion and 1 mL was removed for filtration and determination of the *T* = 0 phage titer. The remaining 5 mL was incubated for 50 min (i.e., one viral replication cycle) at 37°C with shaking and then filtered for phage quantification. Subsequent cycles were initiated by transferring 600 μL of filtrate to a fresh 5 mL culture of relevant *lic2A* phase variant. Five cycles were conducted per day with phage-containing filtrates being stored overnight at 4°C. Each experiment consisted of 20 cycles and five replicates were performed for each cycling pattern. The S100 and R100 cycling patterns consisted of passage in only either ON or OFF populations, respectively. The other cycling series had a two-, three- or four-repeated pattern as follows:- S66, ON-ON-OFF; S50, ON-OFF; S75, ON-ON-ON-OFF; R50, OFF-ON; and R66, OFF-OFF-ON.

### Mathematical Model and Simulations

We describe bacteria-phage interaction using the conventional modeling approach (Cairns et al., 2009; Krysiak-Baltyn et al., 2016). Dynamics of bacterial and phage densities in each experimental cycle (transfer) of number *n* within time T_0_ = 40 min (i.e., before the start of mass replication of phages) is given by

$$\begin{array}{lll} \frac{dB_{0}}{dt} & = & -KPB_{0},\\ \frac{dB_{i}}{dt} & = & KPB_{i-1}-KPB_{i},i=1,\ldots ,N-1\\ \frac{dB_{N}}{dt} & = & KPB_{N-1},\\ \frac{dP}{dt} & = & -PK\overset{N-1}{\underset{i=0}{\sum }}B_{i}-mP,nT<t<Tn+\;{\text{T}}_{0}, \end{array}$$

where B_0_ is the cells/mL of phage-free bacteria; B_*i*_ is the cells/mL of bacteria with *i* phage attachments; *P* is the pfu/mL of free phages. The maximal number of phage attachments for an individual bacterial cell is given by *N*. In the model, we assume that the injection rate is very fast (i.e., instantaneous), so that attachment of one phage immediately results in an infection.

For simplicity, we assume that there is no bacterial growth. We also assume that all phage attachments occurring within the first 10 min lead to replication (each phage produces *b* new phages) whereas later attachments result in phage loss without replication. We neglect binding of newly replicated phages within the last Δ = 10 min of each cycle. The other model parameters are: *K*, phage adsorption constant (note that this constant is assumed to be independent from the number of bound phages and that *K* = 0 for phage-resistant bacteria); *m*, natural mortality of phages; *b*, phage burst size.

At the start of each experimental cycle (i.e., just after dilution) all bacteria are phage-free and their number is always equal to B_*S*_, in other words,

$$\begin{array}{l} B_{0}\left(t={\left(Tn\right)}^{+}\right)=B_{s},B_{i}\left(t={\left(Tn\right)}^{+}\right)=0,\\ \;=0,i=1,\ldots ,\;\text{N} \end{array}$$

Here the symbol “+” denotes the time just after the *n*^th^ dilution, i.e., just prior to the cycle (*n* + 1); the symbol “–” denotes the time prior to the *n*^th^ dilution, i.e., at the very end of cycle *n*.

The phage titer is obtained from the final titer in each cycle multiplied by the dilution coefficient C_*n*_ in cycle #*n* (this value can vary between experiments).

$$\begin{array}{l} P\left(t={\left(Tn\right)}^{+}\right)=P\left(t={\left(Tn\right)}^{-}\right)C_{n}. \end{array}$$

The titer of phages just prior to dilution *n* (i.e., at the end of cycle #*n*) is determined by

$$\begin{array}{l} P\left(t={\left(Tn\right)}^{-}\right)=\overset{N}{\underset{i=1}{\sum }}B_{i}\left(Tn-T_{0}\right)b+P_{S}\left(t={\left(Tn\right)}^{-}\right), \end{array}$$

where P_*S*_ are non-attached phages that survived to the end of the cycle; i.e., the phage number at the cycle end, prior to dilution, is given by the number of infected bacteria at time *Tn*–T_0_ multiplied by the burst size, *b*, plus the number of surviving phages P_*s*_.

Susceptible bacteria are characterized by *K* > 0, whereas for resistant bacteria we have *K* = 0. The value of *K* is kept constant across each cycle of 50 min.

Model parameters and verification were derived from experimental settings or findings. Direct observations indicated that B_*S*_ = 1.75 ± 0.25^*^10^8^ cells/ml, *T* = 50 min, and T_0_ = 40 min. The adsorption constant of *K* = 7 ± 3^*^10^−10^/phage/mL/cell/min) was estimated from an adsorption assay (Figure S1). Other parameters were estimated directly from the oscillating prey assay by model fitting (see Figure S2): *b* = 42 ± 5; and *m* = 0.006 ± 0.0031/min. The parameter *N* had a minor effect in our computation and hence we utilized *N* = 20 in all subsequent models.

Further simulations considered the phage-bacterial dynamics across 105 cycles. Variations in the dilution rate *C* were simulated by changing the value of C according to *C* = C^*^(1 + ε), where ε is a normally distributed random variable with a mean of 0 and variance of 0.3^2^. In each cycle, *K* was randomly switched from *K* = 7^*^10^−10^ (susceptible bacteria) to *K* = 0 (resistant bacteria). The frequency of switching was determined by the probability *p*, which gives the probability of encountering susceptible bacteria (e.g., *p* = 0.75 indicates that 75% of the populations are susceptible bacteria). Numerical simulation was based on the standard Runge-Kutta integration method of order 4 using MATLAB software. When the phage titer dropped to or below the low value threshold of P_0_ = 100, we considered that this was equivalent to *P* = 0 and stopped further simulations. The initial titer of phages at time *t* = 0 was 4.28^*^10^7^ PFU/mL.

### Spatial Oscillating Prey Assay

Allocation of each phase variant (i.e., Rd 30S or Rd 30R) to specific wells was determined by numbering wells from 1 to 631 followed by randomization of these numbers into two sets (see Figure S3). One phase variant was added to the first set of numbered wells and the other phase variant to the second set.

Rd 30S and Rd 30R were sub-cultured to OD_600_ 0.1 and then 250 μL of appropriate culture was added to the starter well. Phage HP1c1 was added at a final titer of ~1 ×10^6^ PFU/mL to this well and the volume was adjusted to 300 μL with fresh sBHI broth. After mixing by tituration, 100 μL was removed for phage titration. The plate was incubated at 37°C with shaking for 70 min (a longer incubation time was required in this miniaturized oscillating prey assay for completion of one phage replication cycle; data not shown). Following incubation, the plate was centrifuged at 1500 × *g* for 4 min to pellet bacterial cells and then 20 μL of supernatant was transferred to surrounding wells (see Figure S4). Remaining supernatant was harvested for phage titration. Newly-inoculated wells received 167 μL of a fresh OD_600_ 0.1 culture of either Rd 30S or Rd 30R, depending on the cycling pattern, followed by repetition of all previously described steps. Ten cycles were performed in the left, right, and downward directions, and 20 cycles in the upward direction (see Figures S3, S4). Five transfers and cycles were conducted each day with the 96-well plates being wrapped in paraffin film and stored at 4°C overnight. This assay was conducted once for each population structure.

### Statistical Analyses

Comparison between the amounts of phage in paired samples from the start (transfer 0) and end (transfer 20) of an experiment (e.g., two time points of a series) were performed using paired *t*-tests. One-way ANOVA with Tukey's multiple comparison was used to compare the phage densities observed between the test area of the spatial oscillating prey assay.

## Results

### Low Numbers of Resistant Bacterial Populations Significantly Restrict Phage Spread and Titer

PV can generate high levels of ON and OFF variants of single genes within individual populations but also has the potential to generate population-to-population variation within a meta-population. For example, *lic2A*-positive *H. influenzae* colonies on agar plates will have most cells in a *lic2A* ON expression state but will also contain small, but significant, numbers of *lic2A* OFF variants. Similarly, an *H. influenzae* meta-population may consist of multiple sub-populations with one sub-set of these populations being *lic2A*-positive and another sub-set being *lic2A*-negative. Such population-to-population variation is observed for *H. influenzae* colonies on agar plates and for *H. influenzae* populations isolated from artificially-inoculated animals and asymptomatic human carriers (High et al., 1996; Weiser and Pan, 1998; Hosking et al., 1999; Poole et al., 2013; Fox et al., 2014). Proportions of ON and OFF sub-populations in a meta-population will depend on levels of selection for each expression state and on “founder” effects that influence the starting state of each sub-population. Thus, any phage invading a bacterial meta-population where there is PV of the receptor must contend with highly variable distributions of resistance and susceptible sub-populations.

**Figure 1:**
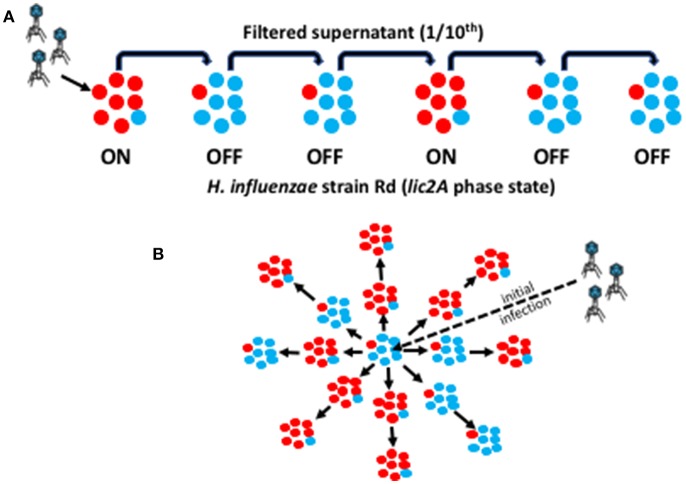
Graphic representation of the linear and spatial oscillating prey assays. This diagram illustrates the methodology for experimental simulation of phage HP1c1 transmission through a linear or spatial meta-population structure. Red circles represent a phage-susceptible Rd 30S cell (*lic2A* ON) while blue circles represent a phage-resistant Rd 30R cell (*lic2A* OFF). An ON population (labeled as ON in **A**) is, therefore, composed mainly of *lic2A* ON cells plus a small number of *lic2A* OFF cells generated by PV during growth of the population with the converse for an OFF population (as indicated by variants present within each sub-population). **(A)** Linear cycling assay for a R66 population structure consisting of one ON/phage-susceptible for every two OFF/phage-resistant sub-populations. Initially, phage are added to an exponential ON phase culture of the bacteria to a final MOI of 0.01 (i.e., ~1 × 10^6^ PFU/mL). After incubation of phage with the host bacteria at 37°C for 50 min, allowing for one viral replicative cycle, the supernatant is harvested and passed through a 0.22 μm filter. An aliquot of 600 μL of filtered culture-suspension is transferred to 5.4 mL (producing a 10-fold dilution of the phage) of a fresh culture of the OFF phase variant. This cycling protocol is then repeated multiple times for a linear pattern of ON and OFF sub-populations. **(B)** Spatial cycling assay for a 66% ON population structure. This assay is performed in a microtiter plate format where wells containing ON or OFF phase-variant sub-populations are randomly distributed across the test area. The ratio of ON:OFF wells describes the structure of the meta-population. We depict an S66 meta-population consisting of 6 OFF/phage-resistant and 11 ON/phage-susceptible sub-populations. The assay is performed in a similar manner to the linear assay except that a reduced bacterial culture volume (i.e., 300 μL) is utilized and passage is to each adjacent population.

To simulate the effect of phage-receptor PV on spread of phage through bacterial meta-populations we developed the linear “oscillating prey assay” (Figure 1). This assay involves continual cycling of phage through *H. influenzae* strain Rd cultures with either a majority *lic2A* ON or OFF phenotype. Each cycle allows for one round of phage replication after which the phage-containing supernatant is recovered, filtered, and transferred to a new culture of either *lic2A* ON or OFF *H. influenzae* cells. During transfer, the supernatant is subject to a 10-fold dilution. This arbitrary dilution factor simulates loss of phage during population-to-population transmission and hence imposes a non-selective bottleneck on the phage population.

**Figure 2:**
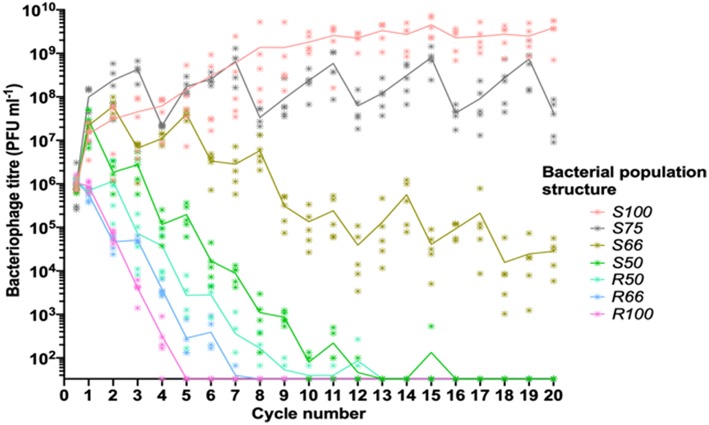
Linear oscillating prey assay for phage HP1c1 infections of *H. influenzae* strain Rd populations with varying population structures for *lic2A* expression. This assay utilizes the format indicated in Figure 1A. Each line represents cycling of the phage through a defined series of cultures of two *H. influenzae* strain Rd phase variants, namely Rd30S (S; *lic2A* ON; phage susceptible) and Rd 30R (R; *lic2* OFF; phage resistant). The population structures are indicated in the legend (i.e., S100, all S; S75, S-S-S-R; S66, S-S-R-S-S-R; S50, S-R-S-R; R50 R-S-R-S; R66, R-R-S-R-R-S; R100, all R). The phage HP1c1 titer was determined at the end of each cycle. Points represent the phage titer observed from each of five biological replicates, with the line showing the mean of these replicates.

A key feature of our experimental model is the utilization of wild-type strains (i.e., Rd 30S and Rd 30R) wherein PV of the *lic2A* gene is occurring. Note that both of these strains also contain the phase-variable type I RM system in an OFF state and switching ON of this system only generates partial resistance to infection. Thus, the phage-resistant *lic2A* OFF populations (derived from Rd 30R) contained a small proportion of ON phase variants that arose during growth of this population at a frequency of ~1 ×10^−3^. Replication of the HP1c1 phage in a *lic2A* OFF population results in production of ~1 ×10^4^ phage due to infection and replication of *lic2A* ON variants in the population (Figure S1). In order to determine whether this low level of phage production influences phage survival, the phage was passaged through only *lic2A* OFF populations (see R100 in Figure 2). Extinction events occurred within 5 cycles indicating that production of phage was not sufficient to overcome the effects of dilution between each cycle for a prolonged period of passaging.

The linear oscillating prey assay was then utilized to test the effects on phage extinction of six population structures with varying combinations of phage-susceptible (i.e., *lic2A* ON; S) and phage-resistant (i.e., *lic2A* ON; R) sub-populations: [1] 100% ON (HP1c1 cycled only through *lic2A* ON populations; S100), [2] 75% ON (3:1 *lic2A* ON:OFF; S75) [3] 66% ON (2:1 *lic2A* ON:OFF; S66), [4] 50% ON (1:1 *lic2A* ON:OFF, starting with an ON culture; S50), [5] 50% OFF (1:1 *lic2A* ON:OFF, starting with an OFF culture; R50), and [6] 66% OFF (1:2 *lic2A* ON:OFF; R66). Survival and propagation of phage was dependent on the proportion of phage-resistant sub-populations in each series of 20 cycles (Figure 2). The starting population had a minor influence on phage survival as shown by the greater number of cycles to extinction observed for the S50 vs. R50 population structures. Survival of phage to the final cycle was only observed when the proportion of phage-resistance populations was ≤ 34% (i.e., the S66, S75, and S100 populations; Figure 2). Extinction events occurred within 7 to 16 cycles in all other heterogeneous populations at a rate that was dependent on the proportion of resistant sub-populations.

Despite survival of phage through to cycle 20 in the S66 population series, phage densities were significantly decreased by this cycling regime (Figure 2; paired *t*-test: *t* = 4.97, *P* <0.05; mean ± SEM PFU/mL values for phage densities at cycle 0 = 7.16 ± 0.26 ×10^5^ and cycle 20 = 2.82 ± 0.9 ×10^4^), indicating that further passages with a similar pattern would have resulted in phage extinction. Contrastingly, the phage titer increased when all sub-populations were phage susceptible (S100, Figure 2; paired *t*-test: *t* = 4.30, *P* <0.05; mean ± SEM PFU/mL values for phage densities at cycle 0 = 9.61 ± 1.15 ×10^5^ and cycle 20 = 3.85 ± 0.89 ×10^9^), with phage titers plateauing after ~11 cycles. Phage titers observed after cycling through the S75 population were also significantly increased in comparison to the inoculum (Figure 2; paired *t*-test: *t* = 2.83, *P* <0.05; mean ± SEM PFU/mL values for phage titers at cycle 0 = 9.71 ± 5.38 ×10^5^ and cycle 20 = 4.29 ± 1.51 ×10^7^). However, the titers produced in this pattern appear to be oscillating, but roughly stable, between cycle 1 and subsequent cycles (S75, Figure 2). Thus, both phage survival and titer were limited by the frequency of encounters with phage-resistant *lic2A* OFF phase variants during passage through a linear series of sub-populations.

### Detection of Combinatorial Effects of Population Structure and a Non-selective Bottleneck on Phage Extinction Using an *in-silico* Model of Phage Spread

**Figure 3:**
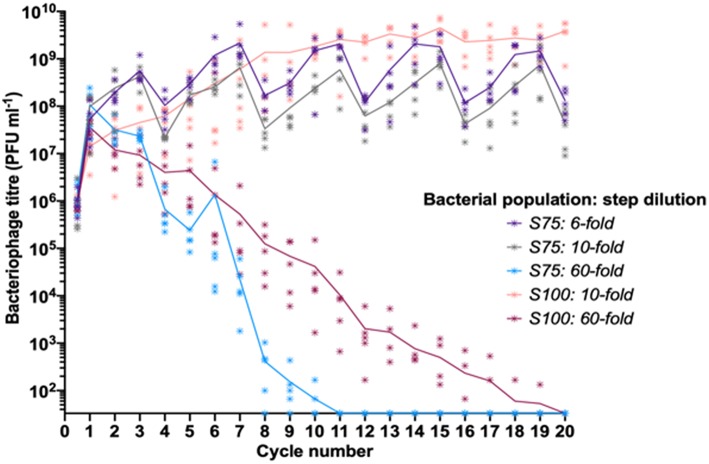
Linear oscillating prey assay for phage HP1c1 infections of *H. influenzae* strain Rd populations with varying dilution constants between each cycle. Each line indicates the mean phage titer during cycling of phage HP1c1 through either the S75 or S100 population patterns when different dilutions are applied between each cycle. The population structures are indicated in the legend (i.e., S100, all S; S75, S-S-S-R), as are the dilution constants applied between each cycle. Both the S75: 10-fold and the S100: 10-fold populations are those also found in Figure 2. The phage HP1c1 titer was determined at the end of each cycle. Points represent the phage titer observed from each of five biological replicates, with the line showing the mean of these replicates.

Our experimental data indicated that differing patterns of S and R sub-populations in a fixed linearly-structured series of populations had a major impact on phage survival and titer over multiple passages. As the outcome of these experiments was likely to depend on the rate of phage loss during transmission between populations, we explored the impact of altering the dilution constant between each cycle of the oscillating prey assay for the two surviving populations from the initial oscillating prey experiments, S100 and S75 (Figure 3). Decreasing the step dilution from 10-fold to 6-fold in the S75 population structure allowed peak phage titers to reach a similar plateau as observed for the S100 population structure with a 10-fold dilution. Increasing the dilution rate from 10-fold to 60-fold between each cycle led to extinction of the phage from both the S75 and S100 populations, with the extinctions occurring at cycles 11 and 20, respectively (Figure 3).

**Figure 4:**
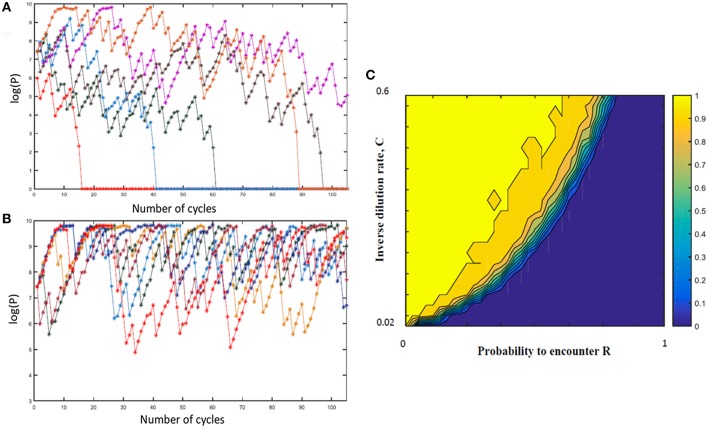
Mathematical model of the impact of sub-population phage resistance/sensitivity composition and dilution rate on phage extinction events. This model simulates transmission of phage HP1c1 through meta-populations of *H. influenzae* strain Rd comprising either phage-susceptible (S, *lic2A* ON) or phage-resistant (R, *lic2A* ON) populations for 105 cycles. In these simulations, the phage is passaged through a linear series of population as outlined in Figure 1 and undertaken in Figures 2, 3. In contrast to the experimental model, the linear order of the S or R populations was stochastic not fixed but with the probability of encountering a resistant population (*R*) being a user-defined value. In this model, the phage population is classified as extinct if the phage titer falls below the extinction threshold (P_0_ = 100) with the titer being set to 0 for all remaining subsequent cycles. **(A,B)** Show examples of model outputs, which are quantified as phage densities log(P). These graphs show six iterations of meta-populations comprising either 75% (i.e., *R* = 0.25; **A**) or 85 % (i.e., *R* = 0.15; **B**) phage-susceptible populations. **(C)** Shows the mean proportion of phage extinction events occurring in 200 lineages for 200 combinations of probabilities to encounter R (i.e., 1 = 100% resistant, 0 = 0% resistant) and rates of phage loss during transmission after each cycle (inverse dilution rate, C; 0.02 = 2% of phage carried through in each transfer). Color correlations are shown on the bar to the right of the graph with 1 representing no extinction events while 0 is extinction in all lineages.

In order to explore a wider range of linear and non-linear cycling patterns and the effects of differing bottleneck sizes (i.e., differing amounts of dilution), we developed a mathematical model of the oscillating prey assay. This model utilized key phage parameters for adsorption rate, replication time, burst size and stability of phage HP1c1 (see Figure S1). In order to simplify model development, the presence of phase variants in each bacterial sub-population was not included as these phase variants only have, as indicated above, a minor effect on phage production. The number of oscillations was extended to 105 phage replicative cycles, while cycling patterns were randomized for each specific overall proportion of *lic2A* ON/OFF sub-populations as a more realistic representation of actual population structures and in contrast to the fixed cycling patterns utilized in the experimental model. The mathematical model produced comparable findings to the experimental setting (Figure S2). Multiple runs of this model exhibited stochastic variation in phage densities and extinction events as observed in the experimental model but with extinction events occurring over a wider band of replication cycles (Figures 4A,B). This model demonstrates that random patterns of ON/OFF expression states for a phage receptor can limit phage spread.

Multiple simulations (*n* = 200) were performed for each combination of R (the percentage of phage-resistant *lic2A* OFF sub-populations) and C (the inverse of the dilution coefficient). Average phage densities were measured for all cycles and runs of each combination of R and C, and phage extinction was defined to occur whenever the density fell below 100 PFU/mL. Phage extinction was always observed when the number of resistant states exceeded 70% of sub-populations, even at a low dilution rate of 1 in 2 (*C* = 0.5; Figure 4C). Similarly, when phage loss was ≥ 98% at each transfer (i.e., 1 in 50 or *C* = 0.02), phage extinction was observed for all meta-population structures including a series of 100% phage-susceptible sub-populations (Figure 4C). Between these extremes, there was an accelerating trade-off between R and C with respect to phage extinction and average phage titer; decreases in dilution rate were countered by a high prevalence of resistant sub-populations enabling bacterial populations to survive even when phage dispersal was low (Figure 4C). This observation suggests that on-going evolution of localized hypermutation of a surface-exposed bacterial epitope could be tuned to the prevalence and amount of phages capable of using the phase-variable epitope as a receptor for surface attachment.

### Localized Hypermutation-Driven Herd-Immunity Produces Regional Variations in Phage Densities Within Bacterial Meta-Populations

Both our experimental and *in silico* oscillating prey assays demonstrated that phage spread was influenced by the linear pattern of phase-variant sub-populations. However, the distribution of phase variants across a surface (e.g., microcolonies on upper respiratory tract surfaces for *H. influenzae*) is anticipated to be random and hence to result in spatial effects on phage spread. Indeed, spatially structured environments are known to restrict phage spread (Brockhurst et al., 2006). Spatial structuring was explored by examining phage transmission across meta-populations of fixed dimensions but with varying proportions of each *lic2A* expression state (Figure S3). This spatial oscillating prey assay was initiated by inoculating phage into one well of a 96-well plate and then expanding outward by seeding each subsequent replicative cycle into neighboring wells (see Figure S4). Phage titers were measured at multiple points across these populations with 40 measurements from the edges providing a limit of detection for the frequency of extinctions of 2.5%.

**Figure 5:**
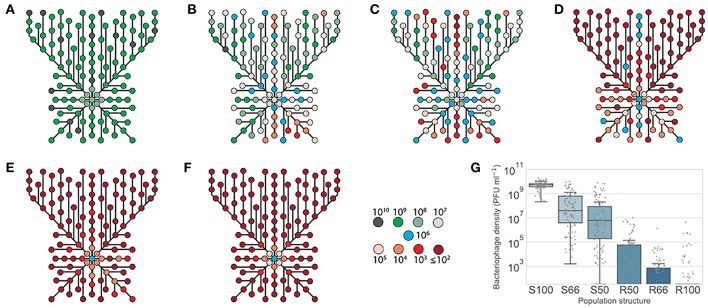
Phage survival in spatially-structured sub-populations of *lic2A* phase variants of *H. influenzae*. Phage HP1c1 was passaged through a two-dimensional array of phage-resistant (R) and phage-susceptible (S) populations as indicated in Figure 1B. The proportion of phage susceptible sub-populations (*lic2A* ON) in each fixed area structure were as follows: **(A)** 100% *lic2A* ON (S100); **(B)** 66% *lic2A* ON (S66); **(C)** 50% *lic2A* ON (S50); **(D)** 50% *lic2A* ON (R50); **(E)** 33% *lic2A* ON (R66); and **(F)** 0% *lic2A* ON (R100). Each node represents a well for which the phage titer was measured. The color of each node indicates the titer of bacteriophage detected in a specific well. Lines indicate the route taken starting from a central initiator well with line length being proportional to the number of cycles between each node. All central initiator wells were inoculated with 10^6^ PFU/mL. **(G)** Shows a box-plot of the distribution of phage densities for each tested well of the fixed-area oscillating prey assays. Densities of phage (PFU/mL) obtained from each test well are represented by a dot for the six distributions of *lic2A* phase variants (x-axis). The boxed area indicates the first to third quartile, the line is the median of all points, and whiskers represent 1.5x the interquartile range. Due to the nature of the small-drop plating methodology employed for phage enumeration the minimum detection threshold at any time point is 3.3 × 10^1^ PFU/mL.

Passage of HP1c1 through heterogeneous populations of the fixed-area oscillating prey assay resulted in differences in mean phage densities between population structures. All population structures contained significantly lower mean phage densities within the spatial area examined compared to the S100 population structure (Figure 5G; one-way ANOVA with Tukey's multiple comparisons; *P* < 0.05). Within populations, uneven phage densities were found across the meta-populations consisting of 50–66% phage-susceptible populations (Figures 5B–D). Conversely, homogenous densities were observed for high (Figure 5A) or low (Figures 5E,F) phage-susceptible distributions. When 66% of populations were phage-susceptible (Figure 5B), densities ranged from 10^3^ to 10^10^ PFU/mL, whereas densities always exceeded 10^8^ PFU/mL if all population were susceptible (Figure 5A). Phage densities in specific regions of heterogeneous populations were dependent on the direction of propagation with passage through multiple resistant or susceptible sub-populations resulting in low or high phage densities, respectively. High extinction rates, as measured using the external wells, were observed in the R50, R66, and R100 populations (82.5, 82.5, and 100%, respectively). This model shows how spatial meta-population heterogeneity could prevent equal dissemination of phage through host populations with phase-variable phage-receptors and aid survival of phage-susceptible sub-populations whose phenotypes may be beneficial for bacterial survival against other selective pressures.

### Observed PV Rates Generate Populations With Phase-Variant Proportions Capable of Herd-Immunity

The spatial oscillating prey assay outputs showed how phage extinction was dependent on the proportion of phage-resistant sub-populations. *H. influenzae* normally resides in the upper respiratory tract where selection is likely to act on both PV states of a locus. Thus, the proportion of resistant populations depends on both selection strength for/against the phage-resistance phenotype, and the ON/OFF switching rate. Switching rates of *H. influenzae* phase-variable genes are malleable due to changes in repeat number and can evolve in response to alternating selection pressures (De Bolle et al., 2000; Palmer et al., 2013).

**Figure 6:**
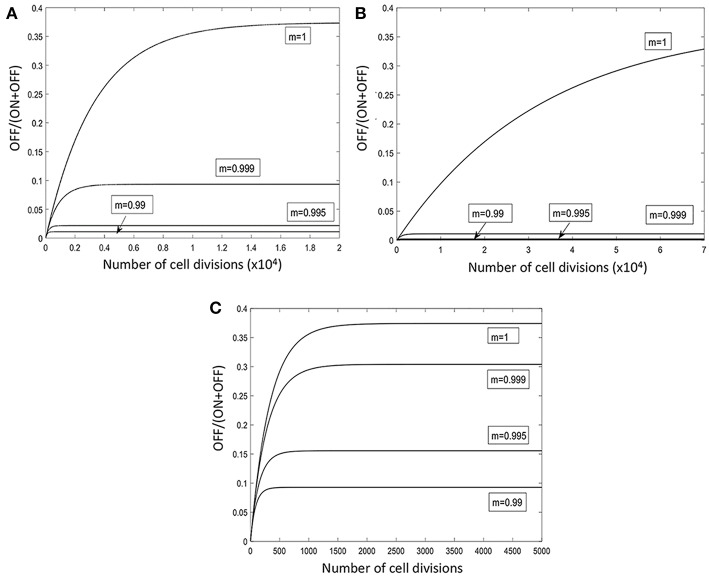
Model of the temporal fluctuations in the relative amounts of phage-susceptible and phage-resistant phase variants for a range of PV rates and selection pressures. PV of the *lic2A* gene results in switching between phage-susceptible (S, *lic2A* ON) and phage-resistant (R, *lic2A* OFF) phase variants. The *lic2A* ON state is however known to mediate serum resistance (see text). This model examines how the rate of *lic2A* PV [α, ON-to-OFF or S-to-R switching; β, OFF-to-ON or R-to-S switching; note that switching rates were obtained from (Dixon et al., 2007)] and the degree of selection (*m*) against the *lic2A* OFF (i.e., R, the phage-resistant state) expression state influences the relative amounts of the OFF/R and ON/S states in a population. All panels show changes in the proportion of the OFF/R state. Three different switching rates were examined: β = 1.89 × 10^−4^, α = 1.13 × 10^−4^ **(A)**; β = 1.89 × 10^−5^, α = 1.13 × 10^−5^ **(B)**; β = 1.89 × 10^−3^, α = 1.13 × 10^−3^ **(C)**.

A mathematical model was developed to examine the impact of different switching rates and strengths of immune selection on the proportions of *lic2A* expression states in a single population (Figure 6; Supplementary Data File S1). Switching rates were selected to mimic the native *lic2A* gene and situations where a gene has a significantly higher or lower PV rate in order to examine whether PV was required to maintain both phage-resistant and phage-susceptible states within a population. Dixon et al. (2007) found that the *lic2A* OFF-to-ON (R-to-S; where R and S are the phage-resistant and phage-susceptible states, respectively) switching rate was 1.7-fold higher than the *lic2A* ON-to-OFF (S-to-R) switching rate. The mathematical model assumed that these proportions were maintained for three 10-fold differences in overall mutation rate representing low, intermediate and high repeats numbers (Figures 6A–C). In the absence of any selective difference between the *lic2A* ON and OFF states (*m* = 1), a high, steady-state proportion (~35%) of the OFF state (i.e., R, the phage-resistant state), was observed for all mutation rates but with minor differences in the rate of approach to the steady state and absolute amounts of this state. The *lic2A* OFF state (i.e., R, phage-resistant state) is known to be more immune sensitive than the ON state (i.e., S), we therefore imposed a selection against the OFF state. Even with strong selection (*m* = 0.99), high levels (8–35%) of the OFF/R state were maintained by high switching rates (Figure 6C). With weak selection (0.999), the OFF/R state was maintained at ~8% but this decreased to 1–5% for stronger levels of selection. In contrast, when switching rates were low (Figure 6B), even weak selection (*m* = 0.999) drives resistant variants to <1% (Figure 6B). Our other *in silico* models indicated that phage spread was inhibited when the probability of encountering OFF/R variants was between 10 and 70% for dilution rates of 0.02 to 0.6. Thus, the immune model shows that observed repeat numbers and switching rates for the *lic2A* gene of *H. influenzae* strains can maintain sufficient phage-resistant variants for restricting phage spread even when there is immune selection against this state.

## Discussion

Hypermutable loci have well-documented roles in facilitating survival of individual bacterial populations against phage predation through generation of phage-resistant cells. An unexplored concept is that hypermutation driven heterogeneity in phage resistance across the wider population also facilitates bacterial survival of phage predation.

### Phase-Variable Loci Can Generate Herd Immunity in Bacterial Meta-Populations

**Figure 7:**
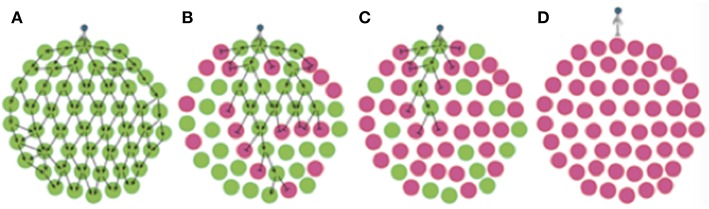
Phase variation of phage resistance genes generates “bacterial herd-immunity” in bacterial meta-populations. Phase variation of a phage-receptor can generate heterogeneous bacterial meta-populations containing a mix of sub-populations that are either susceptible or resistant to phage infection. High proportions of resistant sub-populations can both hinder phage spread and protect susceptible populations from phage attack. In this figure, we show the routes of phage dissemination through four different bacterial meta-populations. Green circles represent phage-susceptible populations while pink circles are phage-resistant populations i.e., *lic2A* ON and OFF populations in our experimental system. Lines with arrowheads show infection events leading to successful phage replication, while lines with bar-heads represent directions where phage-resistant populations act as a barricade blocking phage spread. Regions free of arrows are those free of phage. Phage spread is shown for: **(A)** a 100% phage-susceptible (*lic2A* ON) sub-population; **(B)** 66% phage-susceptible (*lic2A* ON) sub-populations; **(C)** 66% phage-resistant (*lic2A* OFF) sub-populations; and **(D)** 100% phage-resistant (*lic2A* OFF) sub-populations.

The concept of herd immunity was derived to explain the protection of susceptible individuals in populations with high levels of immunity to an infectious agent as observed for measles in Baltimore (Hedrich, 1930). Ordinarily applied to naturally or vaccine-acquired immunity to infectious agents in human populations (Brisson et al., 2016), we show, herein, that repeat-mediated PV of a phage-receptor provides a form of “bacterial herd-immunity” at the population level (Figure 7). The potential for herd immunity in bacterial populations was recently reported by Payne et al. (2018). These authors generated *Escherichia coli* populations containing mixtures of T7 phage-susceptible and –resistant cells where immunity was mediated by a CRISPR-Cas Type II system and a phage-specific spacer. These authors showed that phage spread was retarded in static populations (i.e., in agar plates) even when only 10% of the population was resistant to infection. Our model differs from that of Payne et al. in that we have investigated a hypervariable mechanism for generating phage-resistant and utilized spatially-separate sub-populations as opposed to a continuous population containing a mixture of resistant/susceptible variants.

In our model we observed that phage spread is retarded by resistant sub-populations creating barriers between the phage and phage-susceptible bacterial sub-populations as shown in one- and multi-directional experimental models (Figures 2, 5), and an *in silico* model with randomized patterns of phage sensitivity (Figure 4). Key features of these models were that: the chance of phage survival was inversely proportional to the linear pattern of resistance faced by the phage population; random distributions of phage-resistant/susceptible populations result in large variations in viral numbers across a meta-population with some regions being completely free of phage; and non-selective bottlenecks during transfer of phage between populations modulates the number of phage-resistant populations required to retard phage spread. Thus, localized hypermutation of a phage-receptor generates herd-immunity whereby phage-susceptible sub-populations can be maintained at high levels within bacterial meta-populations.

PV of phage receptors or RM systems is an established phenomenon observed across numerous species and occurring by multiple mechanism. Repeat-mediated switching due to hypermutation of polyG tracts in phage receptor genes of *C. jejuni* strain NCTC11168 abrogates infection by phage F336 (Sørensen et al., 2011). Similarly polyG hypermutation controls the phage growth limitation system of *Streptomyces coelicor* A3(2) and confers resistance to infection with phage φC31 (Sumby and Smith, 2003). Epigenetic PV of Ag43 in *E. coli* or the gtr^P22^ operons that control O-antigen modification in *Salmonella* are known or proposed to modulate phage infection (Gabig et al., 2002; Broadbent et al., 2010). High frequency, but not hypermutable, mutations in short polyG or polyA tracts produce resistance to phages in both *Bordetella pertussis* (Liu, 2002) and *Vibrio cholerae* (Seed et al., 2012). Although these mechanisms vary in the rates of generation of phage resistant variants and in the strength of phage resistance (e.g. high for receptor PV and low for RM PV), they all have the potential to generate spatially-structured populations and hence herd immunity to phage infection.

Additionally, there is potential for evolution of the herd immunity state. PV rates can evolve through changes in the mutable mechanism. Thus, repeat-mediated PV rates increase as a function of repeat number. We anticipate that frequent exposure to phage would select for a greater capacity to form heterogeneous meta-populations through secondary selection for an increase in mutability of the phage receptor.

### Evidence for Immune-Driven Selection of *lic2A* ON Expression States

One rationale for the existence of PV-driven herd immunity is that protection of the phage-susceptible state is required because this state is advantageous under certain circumstances. For *lic2A*, the ON state in *H. influenzae* strain Rd is phage-susceptible and this states leads to extension of the LOS side-chain from the third heptose with a single galactose, a digalactose or more complex sugars. In *in vitro* studies, *lic2A*-dependent epitopes aid in survival against human immune responses (Clark et al., 2013), with the LOS extensions associated with *lic2A* expression encoding epitopes also present on the human P blood group antigens (Virji et al., 1990). Although human volunteer studies of colonization with *H. influenzae* have found that expression of *lic2A* was not essential for human nasopharyngeal colonization (Poole et al., 2013), the *lic2A* ON state has been associated with disease states, including non-typeable *H. influenzae* pneumonia (Weiser and Pan, 1998). We performed an analysis of 104 *H. influenzae* genome sequences and found that *lic2A* is in an ON state in ~63% of all isolates and is predominantly in the ON state for multiple disease conditions (Figure S5). These results indicate that PV of *lic2A* occurs across a wide range of niches for *H. influenzae* but suggests that selection for the ON state is non-existent or is countered by selection for the OFF state as this proportion is similar to that observed in our model in the absence of selection (Figure 6). However, it is known the SSR tracts contract in the absence of selection (Palmer et al., 2013) and, as all these loci contain multiple 5′CAAT repeats indicative of a high PV rate (data not shown), there is an indication of selection for the ability to switch *lic2A* expression states. While these observations are inconclusive, there is some evidence for a scenario where phage drive evolution of hypermutability rates as the *lic2A* gene oscillates between selection for/against the immune-resistant/phage-susceptible and immune-sensitive/phage-resistant states. A caveat to these conclusions is phage-specificity as phage HP1c1 may be specific to extension of the third heptose of *H. influenzae* LOS whereas Lic2A can, in the appropriate genetic context, generate extensions from any of the three heptoses of the LOS inner core. Further work is required to determine whether HP1c1 is specific for extension from the third heptose and if other phages can target this epitope in *H. influenzae* strains where extension is from the first or second heptose.

### Transmission and the “Phage Loss” Phenomenon

Our mathematical model of herd immunity indicates that phage spread is interdependent on population structure and the rate of phage loss from the environment by a “dilution” bottleneck. Thus, when dilution rates are high, phage extinction events are frequent despite high levels of sensitivity within the bacterial population. While natural rates of phage loss from respiratory environments is unknown, a number of factors have the capacity to impact phage loss, such as humidity, salinity and immune responses (Ehrlich et al., 1964; Dubovi and Akers, 1970; Trouwborst and de Jong, 1973; Łusiak-Szelachowska et al., 2014). For phage infections of human commensals or pathogens, such as *H. influenzae*, more extreme environmental selection pressures will apply as phages are transmitted between carriers of target bacterial species.

There are two potential extreme scenarios where the herd immunity model is applicable and “phage loss” is either low or high. These scenarios are elaborated for *H. influenzae* but are relevant to other bacterial commensals/pathogenic bacteria, and even environmental bacterial meta-populations consisting of multiple spatially-separated sub-populations wherein diffusion of a lytic phage can occur between adjacent sub-populations. Firstly, colonization of asymptomatic carriers by *H. influenzae* is likely to involve a series of microcolonies, a meta-population, distributed across the nasopharyngeal surface rather than one continuous population. Phage will therefore have to transmit between microcolonies thereby imposing a low potential for phage loss such that only high numbers of phage-resistant populations will provide protection for any phage-susceptible microcolonies. A second scenario is a population of *H. influenzae* carriers, in this case phage transmission between carriers is likely to result in significant phage loss and hence low numbers of carriers colonized by phage-resistant populations could prevent phage spread to all carriers. These scenarios illustrate the central role of transmission in shaping bacterial herd immunity and in the impact of this fitness trait on localized hypermutation of the phage receptor.

### Bacterial Herd Immunity Could Impact on Phage-Driven Evolution

We observed that phage densities were highly variable across spatially-structured bacterial populations with some regions exhibiting a complete absence of phage (Figure 5). This imposition of spatially-discrete levels of phage selection could select for alternative adaptive traits within the bacterial host. In studies of *Caulobacter crescentus*, low phage selection led to isolation of >200 phage resistance mechanisms, while only ~60 distinct resistance forms were isolated during high phage selection (Christen et al., 2016). Thus, bacterial herd-immunity may prevent uniformity in phage selection pressures across bacterial meta-populations leading to evolution of distinct phage-resistance mechanisms within a single clonal lineage.

### Implications of Lytic and Lysogenic Life Styles

The *H. influenzae* HP1c1 phage has the capacity to enter into a lysogenic state. It is possible that entry into the lysogenic state may overcome the restrictions arising from PV of the receptor both in individual and meta-populations. Future iterations of our computer model will seek to test how lysogenic conversion, and occasional reactivation, may impact on the dynamics of spread of this phage in a meta-population where herd immunity is occurring as outlined herein. A possible suggestion from these ideas is that lytic and lysogenic phages may be impacted differentially by PV within a meta-population with lytic phages being more susceptible to going extinct. Future studies could evaluate the extent to which phages of both these life-styles target phase-variable and non-phase-variable receptors or are impacted by phase-variable RM systems.

### Limitations of Our Experimental and *in silico* Models and the Requirement for Further Testing

Our experimental and *in silico* models assume that phage expansion occurs without mixing and without re-infection. Our assumption of linear spread from a specific focus is a simplification of actual environmental behaviors and requires further testing to determine how mixing may influence the herd immunity phenomenon. However, our approach captures the essential features of phage spread from a microcolony on the mucosa where the nearby microcolonies are likely to be exposed to the highest amounts of phage particles or phage spread from a specific carrier of the host bacterial species to other carriers where there will be linear series of transfer events (albeit with dynamic mixing of the carriers). Re-infection was not considered in our model as we assumed that the surviving population was either killed by the phage or had become fully resistant but could be explored by considering the counter selection for the phage susceptible state over time. We note that the *in silico* model does not completely replicate the experimental model. Thus, no phage extinctions were observed in the experimental model for the S75 linear population structure over 20 cycles whereas extinctions were frequent in the *in silico* model with a randomized S75 population structure with one occurring before cycle 20. Differences may arise due to a combination of stochastic variations in biological and experimental parameters (e.g., perturbations of phage adsorption times, limits of phage detection, accuracy of dilutions, etc.) in comparison to the precision of mathematical values resulting in slightly differing outcomes. Introduction of uncertainty into the mathematical model and further experimental testing may be required to improve the mathematical predictions of outcomes, however these alterations are unlikely to alter the essential relationship (see Figure 4 and relevant text) between population structure, dilution rates and phage extinction as predicted by our models.

### Overall Summary

In summary, our demonstration of a hypermutable locus retarding spread of an infectious agent within a prokaryotic meta-population suggests that the herd immunity phenomenon may be applicable to a wide variety of interacting biological organisms and have deep evolutionary roots. Our conceptual framework could be utilized to explore whether somatic hypermutation, a key example of localized hypermutation in eukaryotes, evolved through selection for sub-population heterogeneity linked to pathogen resistance.

## Data Availability

The raw data supporting the conclusions of this manuscript will be made available by the authors, without undue reservation, to any qualified researcher.

## Author Contributions

CB conceived the starting concept and CB, MC, CT and AM contributed to experimental design. CT performed all the experimental work and bioinformatics experiments. AM generated the mathematical models and performed multiple simulations with these models. All authors contributed to writing of the manuscript.

### Conflict of Interest Statement

The authors declare that the research was conducted in the absence of any commercial or financial relationships that could be construed as a potential conflict of interest.

## Supplementary Material

Raw data from the experimental section of the current work can be found at: https://doi.org/10.6084/m9.figshare.c.4510142.v1
