# Supplementary material for: Phage-Resistant Phase-Variant Sub-populations Mediate Herd Immunity Against Bacteriophage Invasion of Bacterial Meta-Populations
Source: Front Microbiol. 2019 Jul 5;10:1473. doi: 10.3389/fmicb.2019.01473 (PMC6625227; doi:10.3389/fmicb.2019.01473)
Supplement: Supplementary file 1 [file Data_Sheet_1.docx]

**Supplementary Data File S1. Mathematical model to determine the dynamics of PV of the *lic2A* gene of *H. influenza* for a range of mutation rates.**

The *lic2A* gene of *H. influenzae* is subject to phase variation (on/off switching) due to mutations in a 5’CAAT repeat tract present within the reading frame. In *H. influenzae* strain Rd, the ON state of the gene correlates with sensitivity (S) to infection with bacteriophage HP1c1 while the OFF state is associated with resistance (R) to infection. In the absence of phage, the relative proportions of the R and S states will reach a steady state determined by the rates of switching i.e. ON-to-OFF and OFF-to-ON. However, the ON state of the gene is associated with resistance to serum mediated-killing of *H. influenzae*, so that in the absence of phage and the presence of serum there will be selection for the ON state of the gene. The degree of selection by serum components acting on *H. influenzae* occurring within upper respiratory tract during normal host colonisation is unknown but is assumed to be weak. The relative proportions of the ON and OFF states of the *lic2A* gene will therefore be determined by the combination of the switching rates and the level of selection. The aim of this model was therefore to determine the relative proportions of the ON/S and OFF/R states for phage infection for a range of different PV rates and selection pressures for S, the phage-susceptible, serum-resistance state, ON state of the *lic2A* gene.

Switching rates for the *lic2A* gene are taken from previously established switching rates (1) where it was estimated that OFF-to-ON switching of this gene occurred at a rate of 1.89*10^-4^ and ON-to-OFF switching at 1.13*10^-4^. The carrying capacity for the upper respiratory tract is estimated at 10^9^ cfu. The bacterial population is assumed to turnover every hour with a generation time of one hour per division.

In the model, *S* and *R* are the densities of non-resistant (susceptible) and resistant bacteria (cell/L), respectively. We assume for simplicity that the length of the cell life cycle is constant. The mortality rate of bacteria is suggested to be concentration dependent. The densities of *S* and *R* at time t (measured in hours) will be given by

$S\left(t\right)=b\left[S\left(t-1\right)\left(1-\alpha\right)+R\left(t-1\right)\beta\right]\exp\left(-b\left(S\left(t-1\right)+R\left(t-1\right)\right)/K\right)$,

$R\left(t\right)=b\left[S\left(t-1\right)\alpha+R\left(t-1\right)\left(1-\beta\right)\right]\exp\left(-b\left(S\left(t-1\right)+R\left(t-1\right)\right)/K\right)m$,

where *b*=2 is the number of cells obtained after each division; *K*=10^9^ is the carrying capacity (the total maximal number of bacteria that the environment can carry); *α* is the probability of transition *S* →*R* (ON-to-OFF) per cell division, *β* is the probability of transition *R* →*S* (OFF-to-ON) per cell division. The parameter m shows a drop in the fitness of *R* with respect to that of *S* due to extra mortality of *R* (we call it selection). We consider that 0< 1– *m* <<1, i.e. m is smaller but rather close to unity. In other words, the fitness coefficients of resistant and non-resistant bacteria are close to each other.

We start computation from the condition that *S*(0)=10 cell/l; *R*(0)=0.

One can see from Fig. 6 that for the same ratio between the probabilities of switching the final proportion of R {*R*/(*R*+*S*)} in a population would strongly depend on fitness. The actual predicted switching rates would result in 1% and 30% of the cells being in the phage resistant state for weak (m=0.99) versus no selection respectively. For fast mutation rates (higher rates of *α, β*), low level selection (m=0.999) would result in 9% phage resistant variants, however low switching rates combined with low levels of selection would drive the levels of this phage-resistant state to ~0.1% (m=0.999).

We can also calculate the equilibrium ratio of resistant bacteria in the system *R*/(*R*+*S*) analytically using the condition that *S*(*t*+1)=*S*(t)=*S*; *R*(*t*+1)=*R*(*t*)=*R*. We obtain

$S/R=\frac{S\left(1-\alpha\right)+R\beta}{S\alpha+R\left(1-\beta\right)}\frac{1}{m}=\frac{S\left(1-\alpha\right)/R+\beta}{S\alpha/R+\left(1-\beta\right)}\frac{1}{m}$,

We denote *ε*=*S*/*R* and obtain.

$$\varepsilon=\frac{\varepsilon\left(1-\alpha\right)+\beta}{\varepsilon\alpha+\left(1-\beta\right)}\frac{1}{m}$$

For the value of *ε* (epsilon), we arrive to the following quadratic equation

$\varepsilon^{2}\alpha m+\varepsilon\left(m\left(1-\beta\right)-1+\alpha\right)-\beta=0$.

One can see that the above equation always has a unique positive solution.

The value of *ε* and *η*=*R*/(*R*+*S*) are related via *η*=1/(1 +*ε*).

**Supplemental references**

1. Dixon K, Bayliss CD, Makepeace K, Moxon ER, Hood DW. 2007. Identification of the functional initiation codons of a phase-variable gene of Haemophilus influenzae, lic2A, with the potential for differential expression. J Bacteriol 189:511–21.
