# Supplementary material for: Phage-Resistant Phase-Variant Sub-populations Mediate Herd Immunity Against Bacteriophage Invasion of Bacterial Meta-Populations
Source: Front Microbiol. 2019 Jul 5;10:1473. doi: 10.3389/fmicb.2019.01473 (PMC6625227; doi:10.3389/fmicb.2019.01473)
Supplement: Supplementary file 2 [file Image_1.pdf]

Fig. S1 Parameter setting experiments for mathematical modelling.

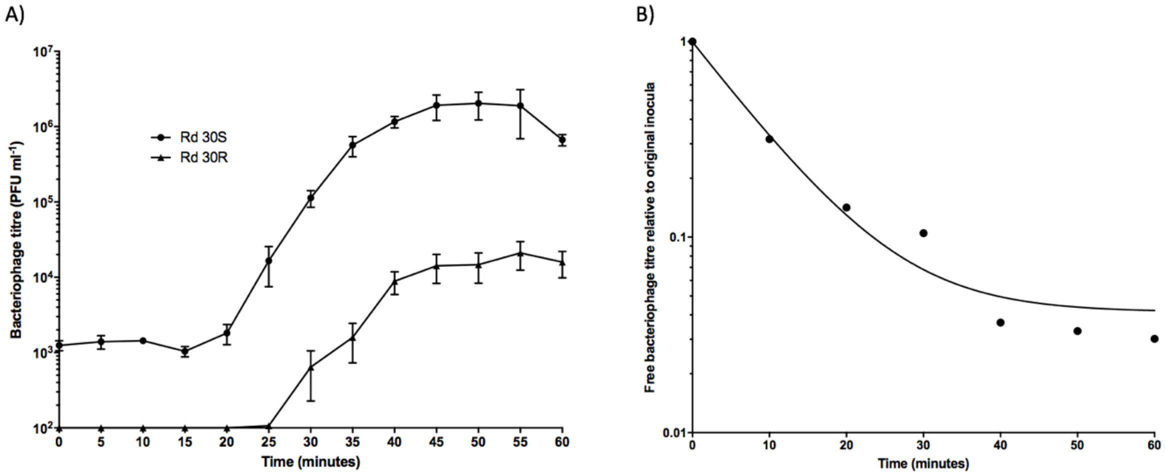

Panel (A) shows a phage one step growth curve used to determine replication time of phage HP1c1 and the burst size of the phage in both phage sensitive and resistant populations. Note that phage replication is still observed in phage resistance populations due to a low level of phage sensitive phase variants where the *lic2A* gene has switched back to the phage sensitive ON state. Figure (B) shows phage adsorption assays for phage HP1c1. The phage was incubated with the phage sensitive *H. influenzae* strain (Rd 30S) and the phage remaining in the media was determined at defined intervals, resulting in a measure of the rate of phage adsorption to host cells. The estimated value of the adsorption constant of phages is K=7±3x10^{-10} ml/(cell min) and was estimated from the linear portion of the curve.
