# Supplementary material for: Phage-Resistant Phase-Variant Sub-populations Mediate Herd Immunity Against Bacteriophage Invasion of Bacterial Meta-Populations
Source: Front Microbiol. 2019 Jul 5;10:1473. doi: 10.3389/fmicb.2019.01473 (PMC6625227; doi:10.3389/fmicb.2019.01473)
Supplement: Supplementary file 3 [file Image_2.pdf]

Fig. S2 Fit of mathematical model to experimental data.

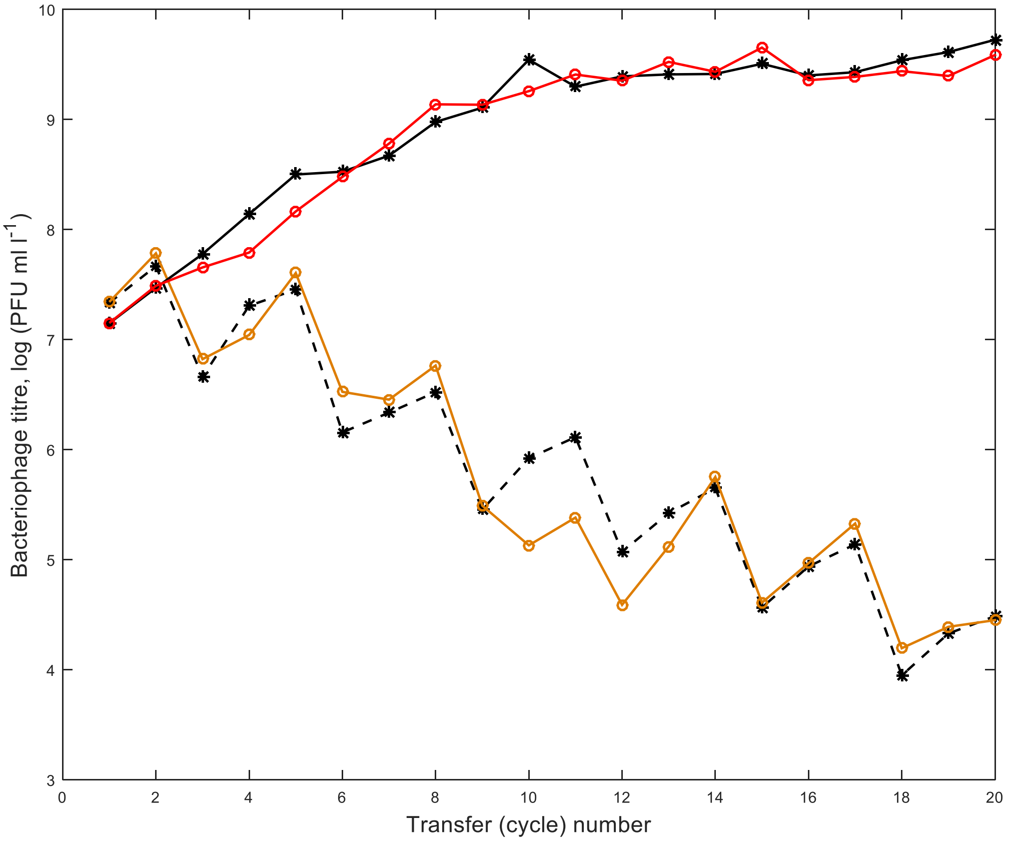

The figure shows the fit of our mathematical model to the oscillating assay data (we plot the phage density at the end of each cycle) for the cases where all bacteria are susceptible (S100%) and where 66% of bacteria are susceptible (S66%). Coloured lines correspond to the experimental data from either the S100 (red line) or S66 (gold line) population structures and the line of best fit from in silico modelling (black line). The estimate of the model parameters are b=42±5 (burst size) and m=0.006±0.0031/min (natural mortality). The other model parameters are given by K=6x10^{-10} ml/(cell min); T=50min; T_0=40 min; B_S=1.75x10^{8} cell/ml; N=20. The values of dilution coefficients C_n were calculated directly from experimental data by taking the ratio of the phage densities before and after dilution.
