# Supplementary material for: Phage-Resistant Phase-Variant Sub-populations Mediate Herd Immunity Against Bacteriophage Invasion of Bacterial Meta-Populations
Source: Front Microbiol. 2019 Jul 5;10:1473. doi: 10.3389/fmicb.2019.01473 (PMC6625227; doi:10.3389/fmicb.2019.01473)
Supplement: Supplementary file 4 [file Image_3.pdf]

Fig. S3 Distribution of wells receiving a *lic2A* ON or *lic2A* OFF phase variant of *H. influenzae* strain Rd for the 50 % ON (A) and 66 % ON (B) population structures.

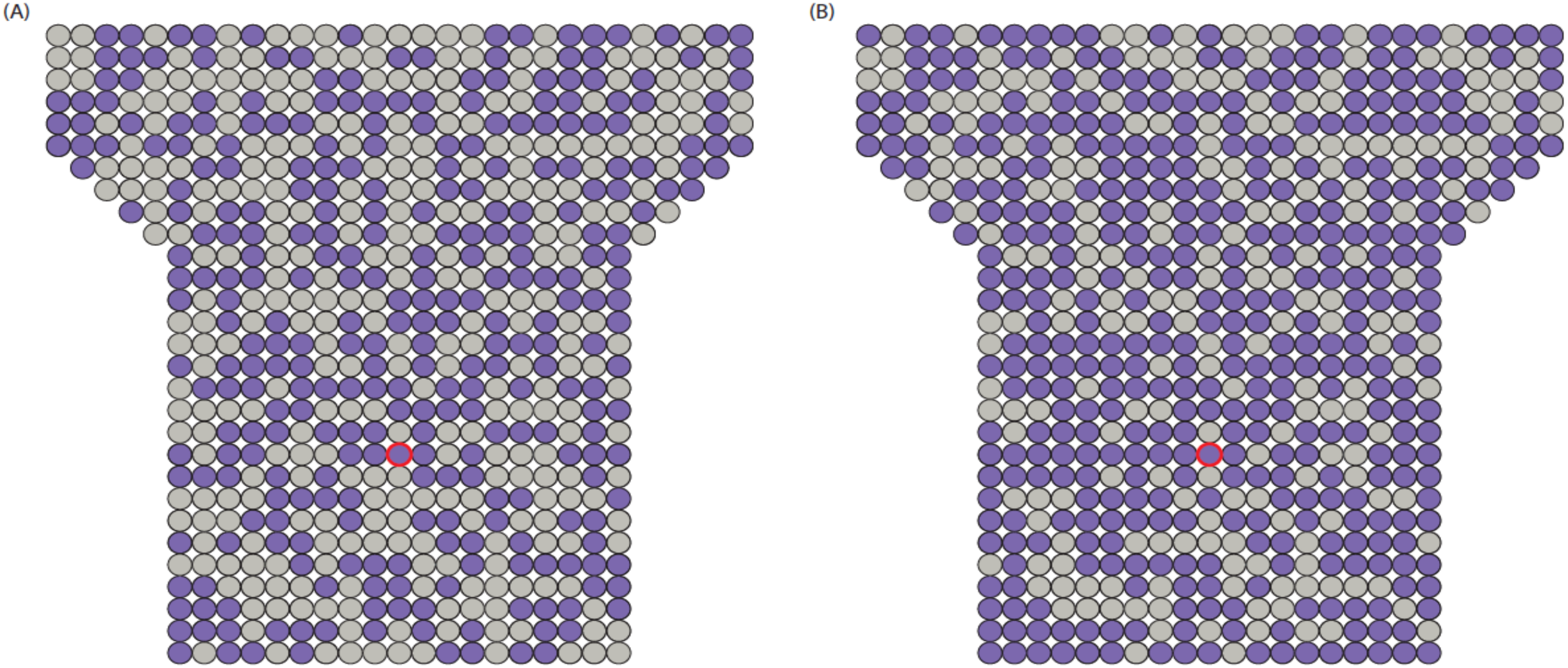

The phase variants of *H. influenzae* strain Rd were grown to mid log phase in BHI broth and this culture was utilised as the inoculum for each well of the spatial assay. Note that these inocula will contain a population in which ~99.9% of the cells are in the expected expression state and ~0.1% in the alternate state due to PV during growth of the culture. In both examples, the filled purple circles represent wells inoculated with a *lic2A* ON population, while grey filled circles represent the *lic2A* OFF populations. For the 50 % OFF and 66 % OFF populations, the fill patterns for the wells were reversed**.**
