# Supplementary material for: Phage-Resistant Phase-Variant Sub-populations Mediate Herd Immunity Against Bacteriophage Invasion of Bacterial Meta-Populations
Source: Front Microbiol. 2019 Jul 5;10:1473. doi: 10.3389/fmicb.2019.01473 (PMC6625227; doi:10.3389/fmicb.2019.01473)
Supplement: Supplementary file 5 [file Image_4.pdf]

Fig. S4 Sampling and transfer regimes for testing phage expansion over a fixed area.

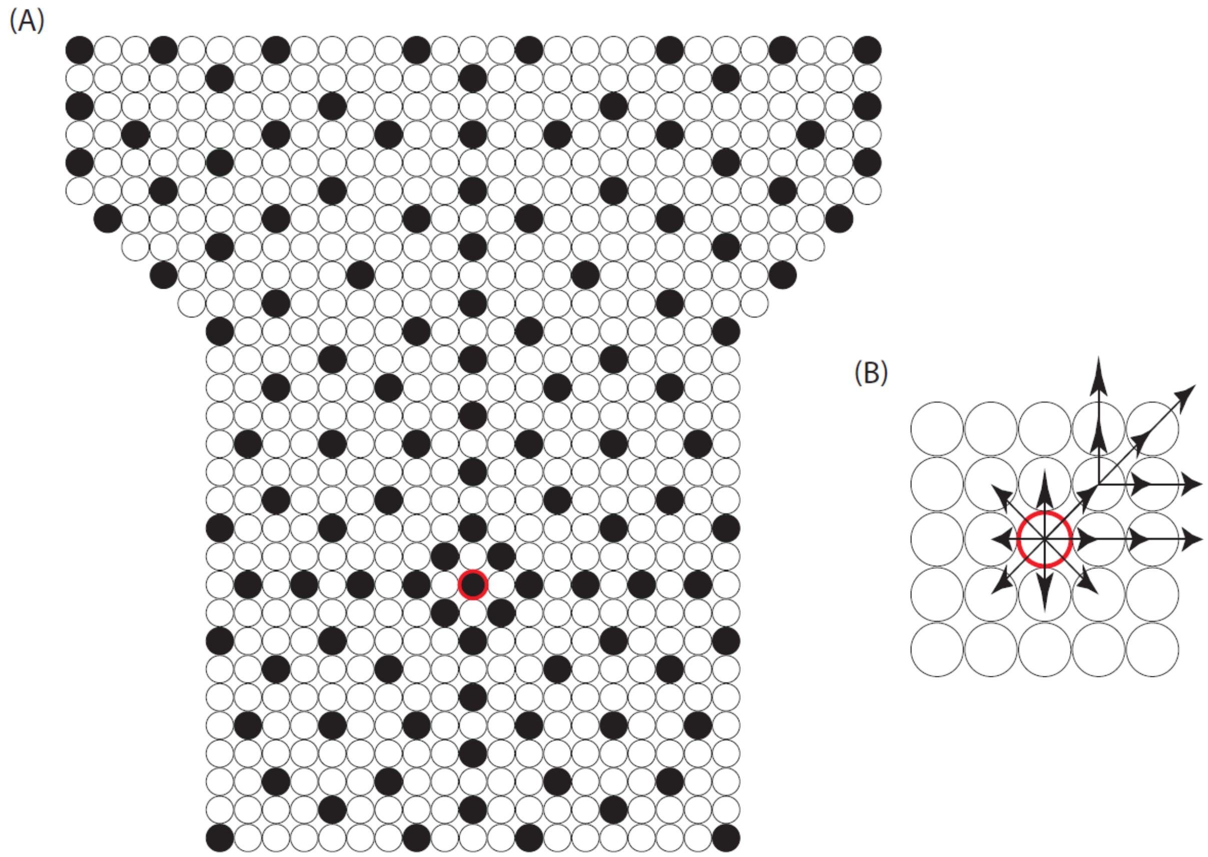

Panel (A) shows the structure of the fixed area over which phage expansion was measured. Each circle represents one well in a 96-well microtitre plate. Filled black circles indicate the wells from which samples were collected from measurement of phage titre. Red circle perimeter shows the well that received the inoculum. Panel (B) shows the pattern of transfer, emanating from the initial well that was inoculated with the HP1c phage, for each sequential cycle of phage infection.
