# Supplementary material for: Phage-Resistant Phase-Variant Sub-populations Mediate Herd Immunity Against Bacteriophage Invasion of Bacterial Meta-Populations
Source: Front Microbiol. 2019 Jul 5;10:1473. doi: 10.3389/fmicb.2019.01473 (PMC6625227; doi:10.3389/fmicb.2019.01473)
Supplement: Supplementary file 6 [file Image_5.pdf]

Fig. S5 Putative ON/OFF state of the *lic2A* gene from 104 *H. influenzae* strains.

(A)

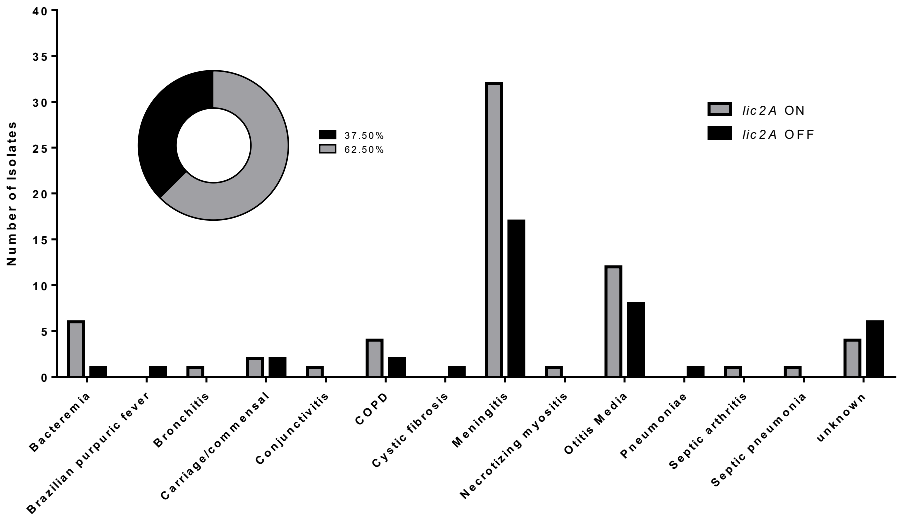

(B)

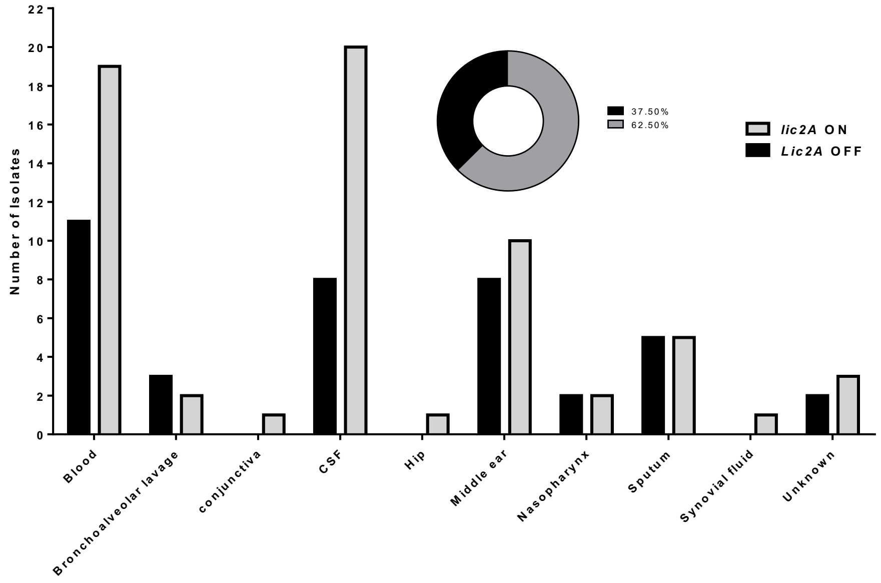

Genome sequences were extracted from the genbank database and searched for the presence of an intact *lic2A* gene. The number of repeats in each *lic2A* were determined and utilised for derivation of the expression state. Panel A is a bar graph of the numbers of isolates with ON (black bars) or OFF (grey bars) *lic2A* expression states for the different clinical states associated with each isolate. Panel B depicts the same data but utilising the site of isolation for each isolate.
